# Application of Adsorptive Blood Purification Techniques during Cardiopulmonary Bypass in Cardiac Surgery

**DOI:** 10.1155/2022/6584631

**Published:** 2022-05-25

**Authors:** Meng-Han Liu, Hong Yu, Rong-Hua Zhou

**Affiliations:** Department of Anesthesiology, West China Hospital of Sichuan University, Chengdu 610041, China

## Abstract

By reason of surgical demand, the majority of cardiovascular procedures still depend on the use of cardiopulmonary bypass (CPB). Due to the nonphysiological state of CPB, it can cause complex and unpredictable inflammatory response, which may lead to significant morbidity and mortality. Unfortunately, the pharmacological and mechanical strategies that currently exist do not offer significant advantages in controlling inflammatory response and improving patient outcomes. The best strategy to reduce inflammation in CPB is still uncertain. In recent years, adsorptive blood purification techniques (BPTs) have emerged, among which CytoSorb is the latest representative device. Currently, the primary application area of adsorptive BPTs is in the control and treatment of systemic hyperinflammatory states, such as refractory septic shock patients. However, the evidences on efficacy and safety of adsorptive BPTs application during CPB surgery are still inconclusive, so we summarize the relevant evidences here and suggest future potential research areas.

## 1. Introduction

Cardiopulmonary bypass (CPB) has been a standard procedure in cardiac surgery since its clinical use in the 1950s [1]. However, it is undeniable that CPB is a known mediator of systemic inflammatory response in both adults and children [2, 3]. Coupled with a series of related adverse clinical outcomes, this pathological inflammation can not be underestimated [4]. For a long time, researchers in different fields have been making unremitting efforts to find various measures to prevent and cure systemic inflammatory response in cardiac surgery in order to reduce its serious harm.

Even with the support of level A evidence, it is almost impossible for a single approach to block multiple pathways of inflammation at the same time [5]. Furthermore, inflammatory mediators are no longer limited to traditional mediators, but more new mediators are involved, such as plasma-free hemoglobin (pfHb) and circulating fragments of the endothelial glycocalyx (EG). Some potential, new, and more effective measures need to be further explored and evaluated. Blood purification technologies (BPTs) have been widely used in dialysis, sepsis, and other fields. In recent ten years, with the emergence of a new adsorptive blood purification device, CytoSorb [6], hemadsorption has been gradually applied during CPB in cardiac surgery for patients at different inflammatory risks, while its efficacy, safety, and potential application need to be further discussed.

## 2. CPB and Systemic Inflammatory Response

### 2.1. Mechanism of Systemic Inflammatory Response Activated by CPB

In the past decades, the essential mechanisms behind the complex pathophysiology of CPB-induced inflammation and organ dysfunction have been largely elucidated [7–10]. The inflammatory response to CPB can be divided into 2 key phases: “early” and “late.” The early phase occurs when blood exposure to nonendothelial surfaces triggers a process called “contact activation,” and the late phase is driven by ischemia-reperfusion (I/R) injury and endotoxemia [7]. During the whole process, five plasma protein systems (contact, intrinsic coagulation, extrinsic coagulation, fibrinolytic, and complement) and five cellular responses (endothelial cells, lymphocytes, monocytes, neutrophils, and platelets) are activated [11]. The most intuitive result is the release of a series of proinflammatory and anti-inflammatory factors. In addition to the well-known interleukin-6 (IL-6, 26 kDa) [12], interleukin-1*β* (IL-1*β*, 17 kDa) [13], tumor necrosis factor *α* (soluble TNF-*α*, 17 kDa; membrane-bound TNF-*α* 26 kDa) [14], and interleukin-10 (IL-10, 18 kDa) [15], new inflammatory mediators such as plasma free hemoglobin (pfHb) [16] and endothelial glycocalyx fragments (hyaluronan, heparan sulphate and syndecan-1) [17] have gradually attracted attention. The overall balance between the two sets of mediators may be more important than the absolute levels of either of them [18, 19]. Once the cytokine response is dysregulated and the body's homeostasis is broken, it will lead to a highly inflammatory state called cytokine storm [6], that is, the term systemic inflammatory response syndrome (SIRS) which has been commonly utilized to define the inflammatory reaction caused by cardiac surgery with CPB [20].

### 2.2. Adverse Effects of Systemic Inflammatory Response Induced by CPB

The main outcome indicators of most studies are limited to laboratory biomarkers, which proves that the level of inflammatory mediators is related to specific organ dysfunction [21]. But clinical studies that take SIRS occurrence as an overall parameter and analyze its impact on clinical outcomes are generally lacking. A retrospective study of 502 patients analyzed the prevalence of SIRS, and its associated clinical impact found that SIRS-positive patients were likely to experience a more complex postoperative course and higher incidence of complications [20]. Hemostasis disturbances [22] and the postoperative cognitive dysfunction [23, 24] after cardiac surgery have also been shown to be a result of the inflammatory response. Severe SIRS has been shown to be associated with higher 6-month mortality [25].

In children, the incidence of SIRS can be 21.9~33.3% or even higher, especially in the presence of low mean age, low body weight, long CPB duration, and large amount of fresh frozen plasma. It is a common complication after pediatric congenital heart disease and significantly prolongs the time of mechanical ventilation and length of PICU and in hospitalization stay [26, 27].

### 2.3. Strategies to Prevent or Mitigate CPB-Induced Inflammatory Responses

Current strategies to prevent or mitigate CPB-induced inflammatory responses have focused on pharmacologic and mechanical therapies. Pharmacologic strategies mainly include glucocorticoids, serine protease inhibitors (aprotinin), phosphodiesterase inhibitors, antioxidants, nitric oxide donors (sodium nitroprusside), complement inhibitors, and sevoflurane [28]. However, not all of these evidence come from powered randomized controlled trials (RCTs). Besides, recent systematic reviews and meta-analyses [29–31] have questioned the prophylactic use of glucocorticoids to reduce mortality.

Mechanical strategies include hemofiltration, leukocyte filters, hypothermia, pulsing flow, and heparin coated circuits [8]. However, no single intervention is supported by strong evidence for clinical benefit [5]. Ideally, the most effective interventions should be able to target multiple inflammatory pathways at the same time [5]. It is necessary to evaluate the comprehensive effect of these existing therapeutic strategies and explore new therapeutic measures in line with the “multiple blows” hypothesis. Over the years, BPTs have developed into an important adjunctive therapy. Based on different principles, BPTs can be divided into the following four types [32]: convection (e.g., HVHF [33], HCO [34]), convection binding adsorption (e.g., coupled plasma filtration adsorption technology [35]), and adsorption type and comprehensive therapy (e.g., renal tubule-assisted and selective cell transplantation devices), respectively. Unfortunately, treatment with convection or dialysis (diffusion) has not been shown to be effective in addressing and controlling cytokine storms to date [36]. Adsorption methods based on the new principle may herald a new dawn for removing excess cytokines and reducing the overwhelming inflammatory response. CytoSorb, the most representative and most recent device of adsorption BPTs, provides a possibility to realize the above expectation due to its nonselective adsorption.

## 3. Adsorptive BPTs

Broadly speaking, hemadsorption refers to the external adsorption of various substances from blood by means of adsorption devices [37]. In a narrow sense, some scholars directly call it extracorporeal cytokine hemadsorption therapy [6]. Adsorptive BPTs are divided into four subgroups: nonselective membranes, semi-selective membranes, selective cartridge, and nonselective cartridges [32], each with its representative devices.

### 3.1. Nonselective Membranes

#### 3.1.1. Acrylonitrile 69 (AN69) Membrane

AN69 (natural AN69), as the first synthetic, high-flux membrane in the world, was originally developed in France in 1969 and introduced for clinical use in 1972 [38]. The polymer used for the AN69 membrane is a copolymer of acrylonitrile and sodium methallylsulfonate. AN69 has the following advantages. First, AN69 is naturally hydrophilic because it contains a large amount of sulfonate groups which can attract water and form hydrogel structure [38]. Second, AN69 has a high adsorption capacity due to the structure of hydrogel and the charge properties of polymer. Third, AN69 is the world's first biocompatible membrane. As early as the 1990s, AN69 was listed as “the most biocompatible membrane and one of those allowing the best clinical tolerance” [39]. AN69ST (surface treatment) hemofiltration membrane is a derivative of AN69. The difference between the two is that the surface of the former in contact with blood reduces its electronegativity by grafting cationic complexes. This allows heparin to bind into AN69ST membrane directly, making it possible to reduce the systemic dose of heparin and even make heparin-free dialysis possible [38].

So far, this kind of membrane has been used mainly in patients with acute renal failure and sepsis. A single-center retrospective comparative study showed that AN69ST hemofilter could be more effective than polymethyl methacrylate (PMMA) hemofilters for improving the 28-day survival outcomes of patients with or without sepsis [40]. However, the two latest studies disagree on the efficacy of AN69ST in sepsis. Hayashi et al. reported that the AN69ST membrane was not superior to the standard CRRT membrane in the treatment of sepsis due to acute panperitonitis [41], while Nakamura et al. suggested that AN69 had a strong adsorption capacity for high mobility group box 1 protein (HMGB1), a typical endogenous risk cytokine in sepsis [42]. What is more, anaphylactoid reaction during hemodiafiltration on AN69 membrane has been observed in both adults [43] and pediatrics [44].

#### 3.1.2. PMMA and Related Membranes

PMMA membrane, a hydrophobic synthetic polymer membrane with microporous structure, is a kind of symmetric membrane with much higher effective exchange area than polysulfone membrane, which can efficiently adsorb medium and large molecular weight molecules [45]. It is high-performance membrane dialyzers that is commonly used as an adjunct to maintenance hemodialysis. There are positive results regarding PMMA applications. PMMA-CVVH treatment significantly reduced tissue and systemic complement activation, limited kidney damage and fibrosis, and effectively regulated immune dysfunction in lipopolysaccharide induced AKI [46]. However, further long-term prospective studies are needed to determine whether PMMA membranes improve outcomes [47]. In addition, the biggest disadvantage of PMMA is easy to condense and needs to be replaced every 24 hours for the duration of treatment [6].

### 3.2. Semiselective Membrane

#### 3.2.1. AN69 oXiris Membrane

oXiris, modified AN69ST, is a hollow fiber structure polymerized by acrylonitrile and methallyl sulfonate, which removes larger molecular weight molecules by membrane binding [48]. Compared with AN69, oXiris uses 3 times polythene imine to enhance endotoxin adsorption and 10 times immobilized heparin to reduce thrombosis [6]. The device was first approved in Europe in 2009, and its indications were expanded in 2017 to include patients requiring blood purification and patients with elevated levels of endotoxins and inflammatory mediators [48]. In vitro trials, oXiris has been shown to be the only device capable of simultaneously removing endotoxins and cytokines, and binding to oXiris was mainly ionic [48]. A case series from Hong Kong reported the positive role of oXiris membranes in accelerating improvement of organ dysfunction [49]. But there have actually been limited clinical trials using the device so far, and in view of this situation, a prospective, national registry on the oXiris membrane has been established in Italy [50].

### 3.3. Selective Cartridge

#### 3.3.1. Polymyxin B Hemoperfusion

Polymyxin B, a multicationic antibiotic, exhibits strong bactericidal activity against Gram-negative bacteria by binding to the lipid A portion of endotoxin and inactivating the endotoxin [51]. To counter the known nephrotoxicity and neurotoxicity of polymyxin B, a polymyxin B immobilized fiber column (PMX) was developed in Japan in 1994, in which polymyxin B was covalently bonded to the surface of polystyrene derivative fibers using the primary amine group of diaminobutyric acid residue [52]. Toraymyxin® (Toray Medical Co., Ltd., Japan) was developed in the same year as a PMX-based medical device to remove circulating endotoxins from human blood together with direct hemoperfusion [53]. The standard blood flow for PMX-HP procedures depends on the column size; for example, PMX-20R is practiced through a whole blood circulation at a blood flow rate of 80 to 120 mL/min [53].

In an in vitro experiment, endotoxin (lipopolysaccharide) removal was most rapid with Toraymyxin compared to oXiris and CytoSorb and the mean adsorptive clearance over the first 30 min was ~20 mL/min [48]. In clinical use, PMX-HP has been safely used for the treatment of septic shock since its development. Cohort studies using large clinical databases still suggest a survival benefit [52]. Currently, more and more studies are focusing on the potential application of PMX-DHP therapy in the treatment of pulmonary diseases such as COVID-19 pneumonia [54], acute respiratory distress syndrome [55], and interstitial lung disease [56, 57].

### 3.4. Nonselective Cartridge

#### 3.4.1. CytoSorb

CytoSorb is a CE-certified commercial blood adsorption medical device. It was first approved in Europe in 2011 and is the only specially approved extracorporeal cytokine adsorber in the European Union [6]. In fact, it can be used either alone or in combination with renal replacement therapy, extracorporeal membrane oxygenation (ECMO) or CPB. Currently, CytoSorb hemadsorption device used in clinical comes from two manufactures, CytoSorb® (CytoSorbents Corporation, Monmouth Junction, New Jersey, USA) and CytoSorb™ (CytoSorbents Europe GmbH, Berlin, Germany), respectively.

CytoSorb has the following unique adsorption characteristics. First, the surface area for adsorption (45,000 square meters) is quite large, which is equivalent to several football pitches and far exceeds that of traditional hemofiltration. Second, the device has strong adsorption capacity and wide adsorption range. CytoSorb consists of a 300 mL cartridge prefilled with a sterile isotonic sodium chloride solution and highly biocompatible porous polymer beads [37], each with a diameter of 300-800 *μ*m, designed to adsorb 5-60 kDa hydrophobic molecules. Within this range, the device can remove a variety of molecules from the blood: proinflammatory and anti-inflammatory cytokines, myoglobin, bilirubin, bile acids, drugs, and so on [58]. However, it does not capture endotoxins [48]. The typical duration of treatment with CytoSorb is up to 24 hours per session, daily for 2-7 consecutive days. And blood flow is set in the range of 150-700 mL/min [59]. Last but not least, the removal of CytoSorb depends on the concentration of the substance [6]; that is, molecules with high plasma levels can be removed more efficiently and faster than molecules with low plasma levels. Due to this internal regulatory mechanism, it is impossible to completely remove substances from the body [37]. But it should be warned that this is not a complete proof of its safety. In an in vitro study, although CytoSorb showed the best cytokine adsorption kinetics, it was precisely because of its nonselective adsorption that CytoSorb lost the most protein and albumin [60].

Two recent reviews [58, 59] of CytoSorb have suggested that adsorption therapy using this device appears to be safe and effective, but larger RCTs are needed to expand our knowledge of the new indication and target population. Hawchar et al. summarized 33 available studies from PubMed database and demonstrated that early hemadsorption therapy plays an important role in rapidly resolving hemodynamic instability in patients with refractory paralytic shock [61], while another updated retrospective study showed that there was no advantage in alleviating cytokine storms or reducing mortality in critically ill patients with CytoSorb treatment compared to matched patients [62].

Overall, from a comprehensive point of view, CytoSorb not only has the widest adsorption spectrum but also is convenient to be installed in the extracorporeal circulation circuit. As the latest adsorptive blood purification device, more and more studies have focused on the clinical application value of CytoSorb in various fields, including patients with sepsis, septic shock, AKI, COVID-19, and ECMO [63–66], and have achieved encouraging results. Here, we focus on the application of CytoSorb adsorbent device in cardiac surgery patients undergoing CPB. More details of all mentioned membranes and cartridges are shown in Table 1.

AKI: acute kidney injury; AN69: acrylonitrile 69; AN69-ST: AN69-surface treated; CPB: cardiopulmonary bypass; CVVH: continuous veno-venous hemofiltration; ECMO: extracorporeal membrane oxygenation; PMMA: polymethyl methacrylate; PMX-HP: polymyxin B hemoperfusion.

## 4. Evidence for the Application of CytoSorb during CPB Surgery

A selective literature search was performed in the PubMed database from January 2016 until 31 October 2021. The items used were as follows: adsorptive blood purification techniques, hemadsorption, hemadsorption, hemoadsorption, blood adsorption, cytokine adsorption, CytoSorb, cytokine storm, systemic inflammatory response syndrome, inflammatory reactions, efficacy, safety, cardiac surgery, and cardiopulmonary bypass. The primary publications found in the database search were then subjected to an internal selection process. First, duplicates were removed. Selection was based on heading, keywords, abstract, publication date, and context. Then, we included studies on the application of hemadsorption in cardiopulmonary bypass cardiac surgery. The intraoperative application mentioned here includes four conditions: (1) preoperative plus intraoperative, (2) sole intraoperative, (3) intraoperative plus postoperative, and (4) continuous application from preoperative to postoperative. Studies were excluded for postoperative application only and excluded for application in patients with inflammatory reactions due to off-pump cardiac surgery or various other nonoperative causes during ICU or hospitalization. Considering the limited number of studies currently available on this topic, we deliberately avoided rigorous assessment of research quality and partially included low-evidence publications (e.g., case reports). The application of CytoSorb in different types of cardiac surgery and different populations is summarized as follows.

Of note, almost half of CytoSorb® and CytoSorb™ are used in the relevant studies involved in this review, and there is no comparative study of the two, so CytoSorb is used to replace the two in the following.

### 4.1. Evidence in Adult Patients Undergoing Elective Complex Cardiac Surgery

There are one systematic review [67], one multicenter RCT [68], five single-center RCTs (one ongoing) [69–73], and three related published studies [74–76]. In the systematic review published in 2021, except two RCTs investigating the application of extracorporeal cytokine adsorption therapy as a therapeutic add-on treatment in sepsis or septic shock, the other five RCTs investigated the application of CytoSorb as a preventive measure in cardiac surgery from 2016 to 2019. The included population of the other nine studies are patients undergoing elective complex heart surgery, including coronary artery bypass grafting, valve replacement, and combined procedures. The CPB time is expected to be at least 90 minutes, or even more than 120 minutes or 3 hours for those with higher requirements. Cytokine levels were the primary endpoint of the five single-center RCTs, and pfHb was the primary endpoint of the multi-center RCT, while all clinical outcomes, hemodynamic parameters, and other laboratory indicators were secondary outcomes.

#### 4.1.1. Inflammatory or Infection Parameters

*(1) Cytokine Levels*. Except for one ongoing RCT [70], there was no significant difference in IL-6 plasma levels in the other four published RCTs with cytokine levels as the primary endpoint. As to IL-8 and TNF-*α*, only one RCT [72] yielded a positive result. In this trial, 40 patients undergoing moderately complex cardiac surgery were randomized to receive either standard care or hemoadsorption treatment with CytoSorb during CPB. The authors found significant reductions in IL-8 and TNF-*α*. However, the positive effects were minor and of short duration, limited to the end of CPB and within 6 hours after CPB. As to IL-10, the first pilot study conducted by Bernardi et al. [69] observed a longer-lasting anti-inflammatory effect of IL-10, but there were high interindividual differences in cytokine levels between their patients.

*(2) pfHb*. A multicenter RCT [68] involving eight academic medical centers was the first to use *pfHb*, a special inflammatory marker causing end-organ dysfunction, as the primary efficacy endpoint. They found that CPB duration combined with a surgical method was more predictive of pfHb levels. The peak pfHb level was the highest in patients undergoing valve replacement surgery, and CytoSorb's ability to reduce pfHb and activated complement levels was more significantly observed in this subgroup. This may be due to the relatively long treatment time (2.5 ± 1.2 h) and the dual cartridge setting.

*(3) Circulating Fragments of the Endothelial Glycocalyx*. There is an ongoing RCT study whose conclusions have not yet been published, but its subset analysis [74] has explored the soluble glycocalyx component (HEP, HYA, and SYN) and the concentration of atrial natriuretic peptide (ANP), a possible glycocalyx abscission promoter, before and after hemoadsorption in 15 patients undergoing CPB cardiac surgery. CytoSorb is proved for the first time to adsorb plasma HEP released during CPB, while ANP and other soluble glycocalyx components (HYA, SYN) cannot be reduced.

#### 4.1.2. Circulating Microvesicles (MVs)

A subgroup analysis [75] of the RCT conducted by Bernardi et al. [69] further investigated the effect of HA on circulating microvesicles, a novel cellular communication network in inflammatory processes, in patients undergoing CPB surgery, but failed to find any effect on circulating MVs count and function by the use of CytoSorb.

#### 4.1.3. Clinical Outcomes

None of the four published single-center RCTs and the multicenter RCT showed significant differences in hemodynamic stability, length of ICU stay, length of hospital stay, mortality, or incidence of AKI, as well as other indicators of clinical outcome. None of the studies included by Goetz et al. [67] reported organ function, and they only found very low-quality inconclusive evidence for effectiveness, because none showed statistically significant differences in mortality, ICU length of stay, and length of hospital stay.

#### 4.1.4. Device-Related Adverse Events

None of the studies found significant differences in adverse events that appeared to be directly attributable to CytoSorb devices. Similarly, a subsequent post hoc analysis [76] of the RCT by Bernardi et al. [69] showed that hemadsorption had no effect on hemolysis. Only two of the RCTs included by Goetz et al. [67] mentioned device-related adverse events but were not sufficient to draw any indicative conclusions.

### 4.2. Evidence in Adult Patients with Infective Endocarditis

The valve surgery mentioned in the above elective heart surgery did not indicate whether they were performed for infective endocarditis or for other reasons, and one study [70] even directly excluded patients with infective endocarditis. There were five studies involving only patients with infective endocarditis, including one RCT [77], two retrospective studies [78, 79], and two case series [80, 81].

#### 4.2.1. Inflammatory or Infection Parameters

A recent RCT reported by Asch et al. [77] attempted to evaluate the effect of prolonged use of CytoSorb (continued for 24 hours postoperatively) in patients with infective endocarditis at high risk of inflammation with potential intraoperative bacterial dissemination. But there were no significant differences in median cytokine levels (IL-6 and TNF-*α*) and infection parameters (CRP and PCT) between the two groups. Neither of the two retrospective studies could obtain cytokine values, but according to the analysis of infection indicators, Haidari et al. [78] found that there was no significant difference among CRP, PCT, and WBC levels, but the recovery rate of these three parameters was significantly accelerated in the hemadsorption group. Santer et al. [79] found that the WBC counts of patients treated with hemadsorption decreased significantly in the first five days after operation. The other two case series did not focus on this outcome.

#### 4.2.2. Clinical Outcomes

*(1) Hemodynamic Stability*. In the RCT study [77], the hemadsorption (HA) group had higher catecholamine and fluid requirements in the early postoperative period. Three observational studies [78, 80, 81] all suggested that the HA group had better hemodynamic stability, while only Santer et al. [79] observed that the HA group had significantly increased demand for norepinephrine, milrinone, erythrocyte concentrate, platelets, and FFP.

*(2) Length of ICU Stay*. As a secondary outcome, in the RCT [77] that included 20 patients with infective endocarditis, all patients in the HA group survived, but the ICU stay was significantly prolonged. The same results were observed by Santer et al. [79] and Kühne et al. [81]. Only Träger et al. [80] found a reduction in median length of ICU stay when compared with a historical control group, but the difference was not significant (median 5 vs. median 7.5 days).

*(3) Mechanical Ventilation Time*. Comparing 39 patients treated with HA with a historical control group of 28 patients, Träger et al. [80] found that more patients treated with HA were able to be weaned from mechanical ventilation within 24 hours after surgery and that patients who failed to extubate had a shorter overall mechanical ventilation duration.

*(4) Mortality*. The retrospective studies of Haidari et al. [78] included 58 patients. In univariate analysis, hemadsorption therapy showed a significant benefit for sepsis-related death, although this benefit disappeared in a multiple regression model, while Asch et al. [77] found no difference in“risk of mortality” scores on the ICU and the ICU and 90-day survival showed no differences in case series by Kühne et al. [81], either.

*(5) AKI*. Although the case series of Träger et al. [80] showed a sustained balance control of postoperative inflammatory response, rapid adjustment of metabolic processes, and stability of hemodynamics, which is consistent with their previous case series reporting post-CPB SIRS patients [82] treated with CytoSorb, however, 16 of these patients required CRRT for high-grade AKI. Subsequently, the case series of Kühne et al. [81] suggested that continued use of HA after surgery may benefit patients developing intraoperatively renal failure. But it should be noted that due to the lack of blank control group and postoperative control group using CytoSorb alone, it is difficult to judge whether the positive effect of this study is due to the combined effect of CytoSorb with CRRT or the independent effect of CytoSorb and whether to use CytoSorb combined with CRRT in the postoperative period remains to be determined. And the study itself is affected by selective bias, because the continued use of CytoSorb after surgery depended on whether there was perioperative renal failure or severe hemodynamic instability or high-grade intraoperative findings such as aortic root abscess. Besides, REMOVE [83], an interventional multicenter RCT, planned to use a group sequential (Pocock) design to evaluate the role of hemadsorption in the prevention of organ dysfunction using changes in mean total SOFA (∆SOFA) scores during preoperative and postoperative care as the primary endpoint.

#### 4.2.3. Device-Related Adverse Events

Santer et al. [79] included 214 patients and used inverse probability treatment weighting (IPTW) to achieve a balanced distribution of baseline features in the two treatment groups. In contrast to the other four studies that did not observe significant device-related adverse events, this study reported an almost four-fold increase in the rate of reoperation due to bleeding in the HA group.

#### 4.2.4. Immune Function

Currently, there is a registered RCT designed to study the efficacy of HA with CytoSorb in improving immune function in patients with infective endocarditis from a new perspective. The goal of RECReATET [84] is to investigate whether cytokine adsorption during surgery can modulate monocyte/macrophage function, key immune cells in patients with severe infection. Monocyte immunity will be assessed by a standardized quantitative flow cytometry assay of MHLA-DR. Data from this study will help answer whether cytokine adsorption may improve or even restore the immune capacity of monocytes and will help explore innovative immunomodulatory strategies, thus providing a new theoretical support for the treatment of critically ill patients.

### 4.3. Evidence in Adult Patients Undergoing Aortic Surgery

From a new perspective on the application of hemadsorption in the comprehensive operation of aortic root: Ross operation (3%) and David operation (7%), Wagner et al. [85] focused on the cross regulation between cytokines and miRNA. By measuring the plasma levels of miRNA in the myocardium, monocyte, and vasculature, it can be inferred whether intraoperative hemadsorption modulates the inflammatory process. They found no difference in miRNA126 and miRNA233 but found that miRNA133a was significantly increased at 3 and 18 hours after surgery, suggesting higher myocardial injury. For secondary outcomes, however, there were no differences in either laboratory parameters (inflammatory mediators) or clinical parameters (vasoactive drug requirements, the length of ICU and hospital stay, and extubation time). This just indirectly explained that the role of hemadsorption in controlling inflammatory response and improving clinical outcomes has not yet been reflected.

Two other studies are observational. The largest cohort [86] included 336 patients undergoing aortic surgery complicated with hypothermic circulatory arrest (HCA) and compared patients treated with HA with the control group using a propensity score model. The results were quite positive. HA significantly reduced the need for vasopressors, blood transfusion, and improved acid-base balance. Another small retrospective pilot study [87] included 16 patients undergoing elective aortic surgery with moderate hypothermic CPB obtained similar results. In addition, this study was the first to observe the effect of HA on improving PF (PaO_2_/FiO_2_) ratio and shortening mechanical ventilation duration.

### 4.4. Evidence in Adult Patients Undergoing Orthotopic Heart Transplantation

In the only observational study [88] to evaluate CytoSorb in orthotopic heart transplantation, CytoSorb treatment was associated with a significant reduction in vasopressin requirement within 48 hours after surgery, reduced frequency of renal replacement therapy, shorter ICU stay, and shorter duration of mechanical ventilation. But they only monitored the dynamics of PCT and CRP and did not consider other mediators of inflammation.

### 4.5. Evidence in Emergency Cardiac Surgery for Patients Receiving Anticoagulation Therapy

Hassan et al. [89] retrospectively analyzed the role of intraoperative CytoSorb adsorption in reducing bleeding complications and improving clinical outcomes in 55 patients receiving ticagrelor and rivaroxaban, which are “nondialysable” drugs with high protein binding for the first time, and reported positive results. They suggested that CytoSorb adsorption is the only way to increase patient safety and reduce bleeding complications in emergency cardiac surgery in patients at high risk of bleeding due to treatment with coagulation-active substances. Mendes et al. [90] reported a case of an 83-year-old woman treated with apoxaban who underwent emergency mitral valve replacement for artificial valve endocarditis and had CytoSorb filters added to the CPB circuit. They observed that the insertion of CytoSorb cartridge into CPB was safe and was associated with rapid correction of avoxaban anticoagulation. Another recent case report [91] also observed that perioperative hemadsorption may be associated with clearance of rivaroxaban at high blood concentrations.

In conclusion, the current evidence of CytoSorb in adult cardiac surgery is insufficient. Even the conclusions from RCTs are inconsistent, which may be related to the small sample size, inappropriate setting of admitting-exclusion criteria, and insufficient duration of CytoSorb intervention. A large number of high-quality, prospective, and large-scale studies are still needed to further explore its efficacy.  Table 2 presents a summary of the original studies on the application of CytoSorb in adult cardiac surgery.

ANP: atrial-natriuretic peptide; CI: cardiac index; CPB: cardiopulmonary bypass; CRP: C-reactive protein; HA: hemadsorption; HEP: heparan sulphate; HYA: hyaluronan; IL-6: interleukin-6; MVs: microvesicles; PCT: procalcitonin; RCT: randomized controlled trial; SAEs: serious adverse events; SYN: syndecan-1.

### 4.6. Evidence of Use in Pediatrics

Tirilomis [92] reported a 15-year-old male who underwent emergency aortic valve surgery for endocarditis. This case showed that the prophylactic blood purification with CytoSorb during CPB might have protective effects in the treatment of pediatric endocarditis.

Perez et al. [93] first described the successful use of CytoSorb in a newborn with refractory vasoplegic and cardiogenic shock needing mechanical circulatory support after congenital heart surgery. Since the newborn weighed only 4 kg, the HA column was used for 72 h (d2-d5). The application of the device was not entirely smooth, with two major problems: first is severe hypotension when the device was attached to MCS (a mechanical cardiac support). Second is a significant increase in vancomycin levels (45.4 mg/L) when CytoSorb was removed, resulting in vancomycin poisoning with acute kidney injury. Overall, the authors concluded that the use of CytoSorb inserted into the in vitro circuit is easy and feasible, even in neonates, as long as some precautions are considered.

In addition to the CytoSorb device mentioned above, there are actually other adsorption devices used in cardiac surgery, but the evidence is few. A randomized controlled trial is currently underway to investigate the effects of oXiris membranes on microcirculation endothelial function and outcomes during CPB (OXICARD Study) [94]. We look forward to more evidence from this study on the application of adsorptive blood purification therapy in cardiac surgery.

## 5. Problems to Be Solved and Potential Prospects

### 5.1. Clear Indications for Treatment

Two well-conducted RCTs [69, 71] have demonstrated that HA by CytoSorb is unlikely to be beneficial in the vast majority of elective cardiac procedures with low to moderate inflammatory responses. This seems to suggest that studies should be directed to high-risk patients [73] (aortic arch surgery with hypothermia arrest and selective cerebral perfusion, infective endocarditis surgery, higher EuroSCORE II, emergency surgery or implanted mechanical circulation support, and heart transplantation patients). Since hemadsorption with CytoSorb is concentration-dependent, it is more likely to demonstrate its therapeutic effectiveness in conditions with systemic hyperinflammation. Albeit a sound theoretical premise [95], however, the conclusions of currently available studies in endocarditis, heart transplantation, and aorta surgery are contradictory. Due to the heterogeneity of the disease and the patient, cytokine activation caused by CPB is uneven that high-risk patients may not always show high levels of inflammation. Asch et al. [77] suggested that cardiac surgery for infective endocarditis per se, independent of the severity of SIRS or sepsis, does not seem to be an indication for HA therapy.

To solve this problem, on the one hand, increasing the sample size may be a feasible method. More importantly, it is time to further clarify the indications for treatment with CytoSorb during CPB surgery, that is, to accurately identify patients who are really in a state of hyperinflammatory response in so-called high-risk patients, preferably to determine the threshold of inflammatory parameters to guide more precise treatment.

### 5.2. Appropriate Treatment Time

The treatment time mentioned here includes the start time, duration, frequency, and end time of treatment. On the one hand, the release of inflammatory mediators is not limited to the CPB period but also affected by the basic state of patients and surgical trauma and so on. It has been reported that peak interleukin levels after cardiac surgery occurred during CPB and 6 hours thereafter [69, 71]. On the other hand, it takes some time for the adsorption device to remove inflammatory mediators. The cartridge should be replaced every 8 hours because of the saturation time of the equipment [77]. However, most studies only applied CytoSorb during CPB, which was much shorter than 8 hours. An in vitro experiment [96] showed that TNF, IL-6, and IL-10 were eliminated rapidly 1 h after circulating through the cartridge at initial concentrations of less than 50%. The effect was most pronounced on IL-6, which was no longer detected after 60 minutes, while TNF was still present after 120 minutes in one-third of cases.

For the reasons mentioned above, it is worth thinking about an appropriate timing match between the production and clearance of inflammatory mediators. Whether to start hemadsorption early in CPB surgery, whether to treat in a continuous manner or in a short time but multiple frequency manners, and whether to continue or stop treatment after surgery all need to be further determined in future clinical practice and research.

### 5.3. Optimal Flow Condition

In the current study, blood flow through the loop of the adsorbent blood purification device ranged from 200 mL/min to 600 ml/min, and the conclusions that have been drawn do not seem to be particularly relevant to the volume of the flow. The reason for such an inconsistent conclusion may be that although a certain flow was initially set, the actual flow was not monitored during the studies. Besides, most of the studies used single cartridges, except for one study [68] that used dual-cartridges. Then, what are the different requirements of parallel double cartridges structure for flow?

### 5.4. Selection of More Clinically Meaningful Endpoints

At present, the main outcomes of most studies are the level of various inflammatory mediators. Less attention has been paid to clinically relevant outcomes [67, 78]. Although these biological indicators have undeniable significance, they also have many limitations. First, there may be measurement bias in the measurement of these biological indicators, and they are not always feasible or convenient for clinicians. Second, these indicators may not truly illustrate the clinical benefits of absorptive extracorporeal BPTs and may, on the contrary, result in a waste of research funds. Therefore, we believe that it is important to consider well-powered studies with patient-relevant endpoints.

### 5.5. Potential Security Risks

Most studies have shown that hemadsorption is safe, even in patients at high risk of bleeding [89–91] undergoing emergency heart surgery. However, Santer et al. [79] suggested that the incidence of reoperation for bleeding was higher in the hemadsorption group (34.0 vs. 7.7%; *P* = 0.011). Therefore, the safety of this new adsorptive blood purification device is not absolute, and there may be other undiscovered security risks that need to be further evaluated.

### 5.6. Lack of Cost-Utility Evaluation

Träger et al. [80] believed that although the use of CytoSorb had some additional costs related to treatment, it did obtain potential clinical benefits. Saller et al. [86] suggested that the cost of the device is offset by a significant reduction in the need for transfusion. Hassan et al. [89] also put forward similar views. Besides, the first analysis to assess the cost utility [97] of intraoperative remove of ticagrelor using CytoSorb versus routine care was conducted in the UK and yielded optimistic results. However, in a recent small retrospective study [79], hemadsorption not only failed to achieve good clinical outcomes but also increased its cost due to the significantly increased rates of reoperation for bleeding and blood product administration in the HA group. In view of the above discussion, it is undeniable that there is a lack of evidence on cost-utility issues, and more systematic research is needed.

### 5.7. Possible Target Treatment Population Other than Routine Application

Adsorptive blood purification devices were originally designed for adults, but studies have focused on their potential role in children with inflammation. At present, there are very limited scientific data on adsorptive BPTs in the pediatric population and even more scarce data in pediatric CPB. Although some case reports and observational studies provide positive evidence for the use of absorptive BPTs in children [92, 93, 98–100], the quality of these evidences is low.

What is more, these devices are designed for adults, so there are certain challenges when it comes to children. For example, optimal blood flow in low-weight patients, length of treatment, and plasma level surveillance of certain drugs and physiologic agents remain to be determined [98]. Selection of the filter in relation to weight, blood heating, patient connection, and dialytrauma are also technical challenges [101]. Otherwise, children may be at high risk of low blood pressure, blood dilution, and cardiac arrest.

## 6. Conclusions

The systemic inflammatory response caused by CPB in cardiac surgery has not been completely solved until now. Adsorptive extracorporeal blood purification technologies, especially CytoSorb, have been shown to absorb various cytokines and other mediators and can be easily placed in the extracorporeal circulation circuit. This review has summarized the available data on the application of CytoSorb in cardiac surgery. To date, data on the use of hemadsorption in cardiac surgery are scarce, and data from existing case series, retrospective studies, and single-center/multicenter RCTs are controversial. However, there is no denying that adsorptive extracorporeal blood purification technology opens a new door for the ongoing fight against CPB-associated SIRS. More prospective, large-sample randomized controlled trials are needed to evaluate the safety and efficacy of this technique in CPB.

## Figures and Tables

**Table 1:** Main details of membranes and cartridges.

	AN69	AN69ST	PMMA	oXiris	PMX-HP	CytoSorb
Characteristic	Nonselective	Nonselective	Nonselective	Semiselective	Selective	Nonselective
Year of development	Flat sheet in 1972, hollow fiber in 1980	Flat sheet in 1998, hollow fiber in 2000	—	2009	1994	2011
Adsorption material	A copolymer of acrylonitrile and sodium methallyl sulfonate	Treated with cationic polymer on the basis of AN69	Polymethyl methacrylate	Polymerized by acrylonitrile and methallyl sulfonate	Polymyxin B bonded to the surface of polystyrene derivative fibers	Divinylbenzene copolymer beads with biocompatible
Cytokines removal	Yes Up to 35 kDa	Yes Up to 35 kDa	Yes Up to 65 kDa	Yes Up to 35 kDa	No	Yes Up to 60 kDa
Endotoxin removal	No	No	Yes	Yes	Yes	No
Main application fields	Dialysis, sepsis	Dialysis, sepsis	Dialysis (PMMA-CVVH)	Dialysis, sepsis	Sepsis, pulmonary diseases	Sepsis, septic shock, AKI, COVID-19, ECMO, CPB

**Table 2:** Literature summary of clinical reports relating to the application of CytoSorb in adult cardiac surgery.

Reference	Study type	Disease or surgery	Number of patients	CytoSorb usage details	Primary endpoint	Results	Adverse effects
Asch et al. [77]	Single-center RCT	Acute infective endocarditis	HA *n* = 10 Control *n* = 10	Single cartridge; intraoperatively and continued for 24 h postoperatively; no flow monitoring	Postoperative course of cytokine levels (IL-6, TNF-*α*, and IL-1b) and infection parameters (CRP, PCT, and leucocytes)	May be useful to equalize higher preoperative levels of CRP and PCT; no advantage in reducing inflammatory parameters or improving hemodynamics	No
Bernardi et al. [69]	Single-center pilot RCT	Elective CPB surgery	HA *n* = 19 Control *n* = 18	Single cartridge; during CPB; 200 mL/min	Cytokine levels (IL-1*β*, IL-6, IL-18, TNF-*α*, and IL-10) within the first five postoperative days	No reduction in proinflammatory cytokines; no benefit on clinical outcomes; longer-lasting anti-inflammatory effect of IL-10	No
Garau et al. [72]	Single-center RCT	Elective on-pump cardiac surgery	HA *n* = 20 Control *n* = 20	Single cartridge; during CPB; 300 mL/min	Serum concentrations of cytokines (IL-8, IL-6, and TNF-*α*) and PCT	Reductions in IL-8 and TNF-*α* and improvement in CI	No
Gleason et al. [68]	Multicenter RCT	Elective complex cardiac surgery	Safety population HA *n* = 23 Control *n* = 23 Efficacy population HA *n* = 20 Control *n* = 18	Dual cartridges; CPB time minus 1 h; 350-600 mL/min	Efficacy endpoint: change in pfHb; Safety endpoint: device-related serious adverse events	No difference in SAEs and 30-day mortality; reductions in pfHb during the valve replacement surgery and reductions in C3a and C5a in the overall efficacy group	No
Haidari et al. [78]	Retrospective, nonrandomized study	Native mitral valve infective endocarditis	HA *n* = 30 Control *n* = 28	Single cartridge; pre+intraoperative or intraoperative; no flow monitoring	The incidence of postoperative sepsis, sepsis-associated death, and 30-day mortality	Reductions in incidence of postoperative sepsis and sepsis-related death, and improvement in hemodynamic outcomes	No
Hohn et al. [74]	A subset of a single-center RCT	On-pump cardiac surgery	*n* = 15 (details not mentioned)	Single cartridge; during CPB; 400 ml/min	Pre- and postadsorber concentrations of HYA, HEP, SYN, and ANP	Reduction in HeP	Not mentioned
Kühne et al. [81]	Case series	Endocarditis	Group 1 (intraoperatively) *n* = 10 Group 2 (intra+postoperatively) *n* = 10	Single cartridge; intraoperatively or intra+postoperatively; intra-op: 300-600 mL/min; post-op: 80-150 mL/min	Inflammation/infection parameters, renal function, lactate plasma levels, catecholamine, and vasopressin demand	Postoperative continuation of treatment might be beneficial in group 2 patients with an obviously more pronounced disease severity	No
Mehta et al. [87]	Retrospective pilot study	Elective aortic surgery	HA *n* = 8 Control *n* = 8	Single cartridge; during CPB; no flow monitoring	IL-6 levels, PCT, leukocyte count, CRP	Reductions in IL-6 and PCT; improvements in respiratory and hemodynamic parameters and ICU and hospital stays	Not mentioned
Nemeth et al. [88]	Propensity score-matched cohort study	Orthotopic heart transplantation	HA *n* = 24 Control *n* = 60	Single cartridge; during CPB; 400-500 mL/min	Hemodynamic stability, postoperative inflammatory response	No difference in inflammatory response; may be beneficial to improve organ function and hemodynamics	No
Poli et al. [71]	Single-center pilot RCT	Elective cardiac surgery	HA *n* = 15 Control *n* = 15	Single cartridge; during CPB; no flow monitoring	Key cytokine levels	No benefits in pro- or anti-inflammatory cytokines; no improvement in relevant clinical outcomes	No
Saller et al. [86]	Propensity score-matched cohort study	Aortic surgery with hypothermic circulatory arrest	HA *n* = 168 Control *n* = 168	Single cartridge; during CPB; 500 mL/min	Catecholamines, acid-base status, transfusion rates	Improvements in hemodynamic stability and acid–base balance; reduction in blood transfusion demand; no significant effects on survival	No
Santer et al. [79]	Single-center retrospective study	Cardiac surgery for infective endocarditis	HA *n* = 41 Control *n* = 200	Single cartridge; during CPB; 500 mL/min	Isotropy and blood product demand during reoperations within the first 24 h	No benefits in improving circulation and organ function	Yes
Taleska et al. [73]	Single-center RCT	Elective complex cardiac surgery	HA *n* = 20 Control *n* = 20	Single cartridge; during CPB; 400 mL/min	Cytokine and complement C5a levels, expression of CD64 and CD163 markers on monocytes, granulocytes, and lymphocytes	No benefits in CI or clinical outcome parameters; effects on the expression of CD64 CD163 by CytoSorb	No
Träger et al. [80]	Case series	Acute infective endocarditis	HA *n* = 39 Control *n* = 28	Single cartridge; during CPB; 200-400 mL/min	Serum IL-6 and IL-8, vasopressor dose, MAP, lactate levels, need for postoperative organ support	Reductions in IL-6, IL-8, and lactate levels; improvement in hemodynamic stability	No
Wagner et al. [85]	Single-center RCT	Complex cardiovascular procedures	HA *n* = 15 Control *n* = 13	Single cartridge; during CPB; 300-500 mL/min	Myocardial, monocyte and vascular miRNA plasma levels	Significantly increased the plasma levels of miRNA-133a	Not mentioned
Wisgrill et al. [75]	Subcohort from a single-center RCT	Elective CPB surgery	HA *n* = 9 Control *n* = 9	Single cartridge; during CPB; 200 mL/min	MVs phenotyping and counting	No effect on circulating MV count and function	Not mentioned
